# COVID-19 Pandemic Stressors and Psychological Symptoms in Breast Cancer Patients

**DOI:** 10.3390/curroncol28010034

**Published:** 2021-01-08

**Authors:** Véronique Massicotte, Hans Ivers, Josée Savard

**Affiliations:** 1 School of Psychology, Université Laval, Québec, QC G1V 0A6, Canada; veronique.massicotte@crchudequebec.ulaval.ca (V.M.); hans.ivers@psy.ulaval.ca (H.I.); 2 Oncology Division, CHU de Québec-Université Laval Research Centre, Québec, QC G1R 2J6, Canada; 3 Université Laval Cancer Research Centre, Québec, QC G1R 2J6, Canada

**Keywords:** anxiety, breast cancer, COVID-19, depression, fear of cancer recurrence, insomnia, pandemic, stressors

## Abstract

Background. The current Coronavirus disease 2019 (COVID-19) pandemic is a highly stressful event that may lead to significant psychological symptoms, particularly in cancer patients who are at a greater risk of contracting viruses. This study examined the frequency of stressors experienced in relation to the ongoing coronavirus pandemic and its relationship with psychological symptoms (i.e., anxiety, depression, insomnia, fear of cancer recurrence) in breast cancer patients. Methods. Thirty-six women diagnosed with a non-metastatic breast cancer completed the Insomnia Severity Index, the Hospital Anxiety and Depression Scale, the severity subscale of the Fear of Cancer Recurrence Inventory, and the COVID-19 Stressors Questionnaire developed by our research team. Participants either completed the questionnaires during (30.6%) or after (69.4%) their chemotherapy treatment. Results. Results revealed that most of the participants (63.9%) have experienced at least one stressor related to the COVID-19 pandemic (one: 27.8%, two: 22.2%, three: 11.1%). The most frequently reported stressor was increased responsibilities at home (33.3%). Higher levels of concerns related to the experienced stressors were significantly correlated with higher levels of anxiety, depressive symptoms, insomnia, and fear of cancer recurrence, *r*s(32) = 0.36 to 0.59, all *p*s < 0.05. Conclusions. Cancer patients experience a significant number of stressors related to the COVID-19 pandemic, which are associated with increased psychological symptoms. These results contribute to a better understanding of the psychological consequences of a global pandemic in the context of cancer and they highlight the need to better support patients during such a challenging time.

## 1. Introduction

The current Coronavirus disease 2019 (COVID-19) pandemic is a highly stressful event and findings suggest that cancer patients may be particularly vulnerable to experiencing psychological distress in the context of the current pandemic [1,2]. The immunosuppression induced by some cancer treatments (e.g., chemotherapy) puts them at an increased risk of contracting viruses and of suffering from complications from COVID-19 compared to the general population [3,4,5,6,7]. In addition, due to physical distancing measures, their social support is likely to be limited in this context [3]. Delays and changes in cancer treatments due to the offloading of several hospital activities deemed non-urgent may also lead to patients worrying about the impact of these changes on their cancer prognosis [3,7]. Very few studies have assessed the psychological impact of the COVID-19 pandemic on cancer patients. Results from a study conducted in breast cancer patients revealed that the disruption of oncology services had a significant impact on COVID-19-related emotional vulnerability as well as on anxiety and depression. In addition, COVID-19-related emotional vulnerability significantly predicted worse anxiety and depression [8]. Another study found that job insecurity related to the pandemic predicted higher depression and worse cognitive functioning in women with breast cancer [9]. However, most of the patients included in these studies were not on active cancer treatments [8,9].

The aim of this cross-sectional study was to examine stressors related to the ongoing COVID-19 pandemic and their relationships with psychological symptoms (i.e., anxiety, depression, insomnia, and fear of cancer recurrence (FCR)) in breast cancer patients undergoing cancer treatments. It was expected that a higher frequency of COVID-19 stressors and greater concerns about them would be significantly related to more severe psychological symptoms. Anxiety, depression, insomnia, and FCR were chosen because they have been found to be elevated in the general population since the beginning of the pandemic [10,11] or because some COVID-19 related stressors appeared very likely to increase them (e.g., delays in cancer treatment leading to increased FCR).

## 2. Methods

### 2.1. Participants

Inclusion criteria were: (a) non-metastatic breast cancer diagnosis, (b) scheduled to receive chemotherapy in the upcoming days/weeks, (c) between 18 and 80 years of age, and (d) able to read and understand French. Exclusion criteria were: (a) metastasis, (b) received chemotherapy in the past, (c) severe cognitive impairment (e.g., Alzheimer’s disease), and (d) severe psychiatric disorder (e.g., psychosis and bipolar disorder).

### 2.2. Procedure

This is a secondary analysis of a larger ongoing longitudinal study. Preliminary results were presented at a research conference in July 2020 [12]. Potential participants were recruited at Hôpital du St-Sacrement (CHU de Québec-Université Laval) in Quebec City, Quebec, Canada. This study was approved by the Research Ethics Committee of the CHU de Québec-Université Laval (#2020-4579) on 5 June 2019. Patients were approached during their mandatory educational session on chemotherapy, which took place prior to their first treatment, and were invited to give written consent to be contacted by phone to assess eligibility criteria. Of the 182 patients approached for the larger study, 79 (43.4%) accepted to be contacted, 56 (70.9%) were eligible, and 43 (76.8%) agreed to participate and signed the informed consent form. Three patients were excluded after the beginning of the study (e.g., development of metastases) and one dropped out after the second evaluation. An amendment was approved on 24 April 2020 by the ethics committee to add our COVID-19 stressors questionnaire to the battery of self-reported scales. The questionnaires were sent electronically to the 39 participants and 36 completed them either during (30.6%) or after (69.4%) their chemotherapy treatments (note that they were still on active treatment, receiving either radiation therapy or hormone therapy). In the Province of Quebec, the shutdown and confinement began on 13 March 2020. Questionnaires were completed between 28 April and 29 May 2020. Hence, at the very beginning of the de-confinement (elementary students and construction workers were the first to return to activity on 11 May 2020). The Province of Quebec is considered the epicenter of the COVID-19 pandemic in Canada, accounting for about half of the cases.

### 2.3. Measures

#### 2.3.1. COVID-19 Stressors Questionnaire

This questionnaire includes 14 items that assess the occurrence of 10 stressors related to the COVID-19 pandemic (yes or no, e.g., difficulty obtaining needed help or social support). For each of the experienced stressors, the degree of concern is rated on a 5-point Likert scale ranging from 0 (“not at all”) to 4 (“extremely”). Two additional items assess the degree to which participants worry about two specific situations related to COVID-19 (i.e., the possibility of contracting the coronavirus, the possibility that cancer may progress or be less likely to be cured due to changes in cancer treatment). The total score of the degree of concerns was obtained by averaging the level of concerns related to each COVID-19 stressor experienced and the two additional items. This questionnaire was developed by our research team and has not yet been validated.

#### 2.3.2. Insomnia Severity Index (ISI)

This questionnaire includes 7 items and evaluates the perceived severity of insomnia (e.g., difficulties falling asleep) for the past two weeks [13]. All items are rated on a 5-point Likert scale ranging from 0 (“not at all”) to 4 (“extremely”) for a total score ranging from 0 to 28, which is obtained by adding all items. A total score of 8 or higher indicates a clinical level of insomnia symptoms. The French-Canadian version was empirically validated among cancer patients [14], with psychometric properties similar to those found in the general population (internal consistency, α = 0.90, [15]).

#### 2.3.3. Hospital Anxiety and Depression Scale (HADS)

The HADS includes 14 items divided in two subscales of 7 items that assess depressive symptoms and anxiety experienced in the past week [16]. The items are rated on a 4-point Likert scale ranging from 0 to 3. The total score ranges from 0 to 21 for each subscale and is obtained by adding their respective items. A total score of 7 or higher indicates a clinical level of depression or anxiety. The French-Canadian version has psychometric properties equivalent to that of the English version (internal consistency, α = 0.89, [17]).

#### 2.3.4. Severity Subscale of the Fear of Cancer Recurrence Inventory (FCRI)

This subscale includes nine items rated on a 5-point Likert scale ranging from 0 (“not at all”) to 4 (“a great deal”) [18]. A score of 13 or higher indicates a clinical level of FCR. The total score corresponds to the sum of all the items. One item had to be reversed prior to the calculation. The original French-Canadian version has excellent psychometric properties (internal consistency, α = 0.95, [18]).

### 2.4. Statistical Analysis

All analyses were conducted using SPSS version 25 with an alpha level set at 0.05, two-tailed. First, the frequency of each COVID-19 stressor was calculated. Kendall’s Tau (τ), which is particularly useful to assess associations between ordinal variables that share tied ranks [19], were also calculated to assess the associations between the number of stressors experienced and psychological symptoms. Finally, Pearson’s correlations were conducted between mean levels of concerns related to the experienced COVID-19 stressors and psychological symptoms.

## 3. Results

### 3.1. Demographic Characteristics and Descriptive Statistics

Table 1 presents the main participants’ demographics at baseline (N = 36) and the descriptive statistics. On average, patients slightly exceeded the clinical cut-off score for insomnia (8.1) and FCR (14.3). A fairly large proportion of the patients reported clinically significant levels of anxiety (44.4%), insomnia symptoms (41.7%), and FCR (52.8%). A minority reported a meaningful level of depressive symptoms (16.7%). No significant difference was observed on these variables whether the questionnaires were completed during or after chemotherapy (*p*s = 0.09–0.60).

### 3.2. COVID-19 Stressors and Correlations between These and Psychological Symptoms

Results revealed that most of the participants (63.9%) experienced at least one stressor related to the COVID-19 pandemic (one stressor: 27.8%, two stressors: 22.2%, three stressors: 11.1%, four stressors: 2.8%, range 0 to 4, see Table 2). Stressors that were associated with the highest degree of concerns were: difficulty obtaining food, medicine, and essentials (reported by 8.3% of the patients, M = 2.7), postponement or cancellation of cancer treatment (19.4%, M = 2.4), changes in cancer care trajectory (11.1%, M = 2.3), and postponement of medical tests (11.1%, M = 2.3). Results showed that a higher number of stressors experienced was significantly associated with greater levels of anxiety, τ(32) = 0.34, *p* = 0.01, depression, τ(32) = 0.33, *p* = 0.02, and insomnia, τ(32) = 0.33, *p* = 0.01, but not FCR, τ(32) = 0.12, *p* = 0.38. A higher level of concerns related to the COVID-19 stressors experienced was significantly associated with greater anxiety, *r*(32) = 0.54, *p* = 0.001, depression, *r*(32) = 0.36, *p* = 0.04, insomnia, *r*(32) = 0.55, *p* = 0.001, and FCR scores, *r*(32) = 0.59, *p* < 0.001.

## 4. Discussion and Conclusions

The present study examined stressors related to the COVID-19 pandemic and its relationship with psychological symptoms in non-metastatic breast cancer patients. About two-thirds of the patients experienced specific stressors related to the pandemic and a higher level of concerns related to these stressors was significantly associated with higher levels of anxiety, depressive symptoms, insomnia, and FCR with moderate to strong correlations. In addition, having experienced a greater number of stressors was significantly related to greater anxiety, depression, and insomnia.

Importantly, the mean level of concerns was between “moderately” and “a lot” for five stressors (mean between 2.0 and 3.0), including three stressors related to changes that occurred in cancer care, one about the difficulty obtaining the needed help and support and one related to difficulty obtaining food, medicine, or other essentials. This is quite significant given that the study was conducted very early in the pandemic. It would be interesting to investigate how the level of concerns evolved later on with delays in cancer care and physical distancing measures getting longer and governmental financial help ending.

Our results are in line with those of Swainston et al. [8] who found that COVID-19-related emotional vulnerability predicted higher levels of anxiety and depression in breast cancer patients. Additionally, the proportion of patients reporting clinical levels of anxiety (44.4%) appears to be higher than what is generally found in this population (i.e., 19%, [20]), while the proportion of patients showing a clinical level of insomnia symptoms (41.7%) and FCR (52.8%) falls in the upper range of rates previously found (i.e., 31% to 59% and 44% to 56%, respectively, [21,22]).

Together, these data suggest that cancer patients are vulnerable toward experiencing significant psychological distress in the context of a pandemic and highlight the need to better support them during that time. Increasing access to professional support offered through web conferencing is particularly relevant given physical distancing measures [23]. Online support groups could also help to circumvent the deleterious effects of a lack of social support that was reported by 19.4% of the patients and which contributes to psychological distress. It is well demonstrated that social support predicts a better adjustment to cancer and a better quality of life [24]. Finally, the postponement or cancellation of medical tests and cancer treatment reported by 11.1% to 19.4% of the patients, respectively, is worrisome given their possible impact, not only on patients’ psychological well-being, but also on their prognosis.

## 5. Study Limitations

The present study is among the very few to evaluate psychological symptoms among cancer patients during the first wave of the COVID-19 pandemic. As the epidemic came unpredictably, studies that were already ongoing at that time, such as ours, offered unique opportunities to provide some information on this question. One strength of this study is the high acceptance rate (92.3%) among patients who were contacted. Some limitations also need to be acknowledged. First, the small sample size limits the findings’ external validity. The generalization of findings is also limited to non-metastatic breast cancer patients receiving chemotherapy. In addition, the COVID-19 questionnaire has not been psychometrically validated. Furthermore, the cross-sectional analysis limits the inferences that can be drawn from this study. For instance, it is impossible to determine whether psychological symptoms increased during the COVID-19 pandemic as compared to before. Longitudinal studies with pre-COVID-19 pandemic measures would be needed to better understand its impact on cancer patients’ psychological functioning. Hence, the results should be interpreted with caution and further studies are needed to replicate them in larger samples and in patients with other types of cancer.

In conclusion, breast cancer patients experience several stressors related to the COVID-19 pandemic and these are associated with increased psychological symptoms. A good proportion of the patients show clinical levels of anxiety, insomnia, and FCR. This study contributes to a better understanding of the early psychological consequences a global pandemic has in the context of breast cancer and could serve as a point of comparison for further studies on subsequent waves of the pandemic. It also highlights the need to better support patients during such a challenging time.

## Figures and Tables

**Table 1:** Characteristics of participants and descriptive statistics (N = 36).

Characteristics	M (SD)	N (%)
Age (years)	53.6 (10.9)	
Time since cancer diagnosis (months)	7.4 (2.7)	
Marital status		
Married/cohabitating		24 (66.7)
Single		5 (13.9)
Separated/divorced/widowed		7 (19.5)
Education		
High school or less		10 (27.8)
College		9 (25.0)
University degree		17 (47.3)
Current occupation		
Working (full/part time)		9 (25.0)
Sick leave		12 (33.3)
Retired		12 (33.3)
Unemployed		3 (8.4)
Annual family income in Canadian dollars		
Less than $20,000		2 (5.6)
$20,001–$60,000		11 (30.6)
$60,001–$100,000		6 (16.6)
$100,001–$140,000		5 (13.9)
$140,001 and more		8 (22.2)
I don’t know or refused to answer		4 (11.1)
Descriptive statistics		
Level of concerns related to COVID-19 stressors (0–4)	1.3 (0.8)	
HADS-Anxiety (0–21, cut-off 7)	6.6 (4.0)	
HADS-Depression (0–21, cut-off 7)	4.0 (2.9)	
ISI-Insomnia (0–28, cut-off 8)	8.1 (6.0)	
FCRI-Fear of cancer recurrence (0–36, cut-off 13)	14.3 (6.4)	

**Table 2:** Frequencies of COVID-19 stressors and degree of concerns related (N = 36).

COVID-19 Stressors and Degree of Concerns If Stressors Experienced	M (SD)	N (%)
Increased responsibilities at home (yes)		12 (33.3)
Degree of concerns (0–4)	1.8 (1.1)	
Difficulty obtaining help or social support needed (yes)		7 (19.4)
Degree of concerns (0–4)	2.3 (0.8)	
Postponement or cancellation of cancer treatment (yes)		7 (19.4)
Degree of concerns (0–4)	2.4 (0.8)	
Changes in their cancer care trajectory (yes)		4 (11.1)
Degree of concerns (0–4)	2.3 (1.0)	
Postponement or cancellation of medical tests (yes)		4 (11.1)
Degree of concerns (0–4)	2.3 (1.0)	
Difficulty obtaining food, medicine, or essentials (yes)		3 (8.3)
Degree of concerns (0–4)	2.7 (1.2)	
Having relatives who contracted COVID-19 (yes)		3 (8.3)
Degree of concerns (0–4)	1.3 (0.6)	
Loss of job or loss of income related to COVID-19 (yes)		2 (5.6)
Degree of concerns (0–4)	0.5 (0.7)	
Working in a place where one is susceptible to contract COVID-19 (yes)		0 (0)
Degree of concerns (0–4)	n/a	
Getting sick from exposure to COVID-19 (yes)		0 (0)
Degree of concerns (0–4)	n/a	
Degree of worry linked to specific concerns related to COVID-19		
Possibility of contracting COVID-19 (0–4)	1.6 (1.2)	
Possibility that cancer may progress or be less likely to be cured due to changes in cancer treatment (0–4)	0.6 (0.8)	

Note. n/a: not applicable. The stressor was not experienced. Thus, the degree of concerns related was not assessed.
